# Nineteen years of radiographic screening: Impact of sepsis and evolution of osteochondrosis dissecans prevalence in Walloon sport horses born between 2004 and 2022

**DOI:** 10.1371/journal.pone.0308304

**Published:** 2024-09-10

**Authors:** Raphaël Van Cauter, Isabelle Caudron, Jean-Philippe Lejeune, Alycia Rousset, Didier Serteyn

**Affiliations:** 1 Centre Européen du Cheval, Mont-le-Soie, Vielsalm, Belgium; 2 Département des Sciences Cliniques des Équidés, Chirurgie et Orthopédie, FARAH, Université de Liège, Liège, Belgium; University of Life Sciences in Lublin, POLAND

## Abstract

Osteochondrosis dissecans is a particularly common developmental orthopaedic disorder in equines. Among its causes, the hereditary component is no longer a matter of debate, and, for several decades, the various studbooks for sport horses have been selecting stallions in order to limit the prevalence of this condition in the population. However, to our knowledge, no study has evaluated changes in the prevalence of osteochondrosis dissecans over time through longitudinal monitoring of a population of sport horses. The study presented here is part of a large screening programme for developmental orthopaedic pathologies in Wallonia (Belgium) and assessed the presence of these lesions over a period of 19 years in the Walloon sport horse population according to constant and standardised sampling and diagnostic criteria. The effects of breeding conditions and infection in foals were also assessed by means of questionnaires. The results showed no significant change in the prevalence of osteochondrosis dissecans in a population of 1099 individuals born between 2004 and 2022. Furthermore, individuals who had suffered from sepsis during their growing period were very significantly predisposed (p < 0.001) to the development of osteochondrosis dissecans compared to a control group, with respectively 14/21 (67%) and 103/364 (28%) of individuals affected. This study suggests that the selection programmes applied to the population studied are not sufficiently strong or adapted to reduce the prevalence of osteochondrosis dissecans in the population over a period of 19 years. Moreover, this study confirms that foals with sepsis and concurrent osteochondrosis dissecans lesions should not necessarily be excluded from breeding programmes on this basis.

## Introduction

Since 1947 and the first description of osteochondrosis dissecans (OCD) in equids by Nilsson, this pathological condition has become a major concern for veterinarians and researchers in the field of musculoskeletal pathologies [1–5]. A succession of studies on the subject have demonstrated the high prevalence of OCD in different populations, ranging from 6.25% in feral horses to 36% in Warmbloods [6–9]. The presence of these lesions, particularly those of the lateral ridge of the femoral trochlea, has a negative impact on the performance of affected individuals, both in terms of career longevity and on their level of performance in the various disciplines (sport and racing), predisposing them to the development of secondary lesions such as osteoarthritis and reducing their commercial value [10–13].

Osteochondrosis is defined as a disturbance of the enchondral ossification process, characterised by the presence of ischaemic chondronecrosis induced by a failure of the vascular supply leading to necrosis of the growth cartilage [3, 4]. This pathological condition appears as irregularities, concavities, heterogeneity (change in radiopacity) of the osteo-articular margins and osteochondral fragments, as well as subchondral bone cysts at epiphyseal and, more rarely, metaphyseal level [14–16]. Its aetiology is multifactorial: genetic, dietary, and traumatic [17–20]. In fact, it has been shown that an excess of starch in the diet, severe mineral imbalances and irregular exercise in foals and yearlings are associated with a high risk of developing OCD [21–24]. However, it also appears that certain lesions may result from septic arthritis and other infections in young foals, causing bacterial colonisation of growth cartilage canals, areas of necrosis and failure of the enchondral ossification process within the epiphyses [25, 26].

The hereditary component of OCD was identified early on in different populations of horses [27–29]. The heritability (h^2^) of OCD has been studied and varies according to the method of calculation, the breed and the location of the lesions [30–33]. In order to reduce its prevalence in the population, several studbooks have introduced a selection system, with only male breeding stock free of this condition being allowed to reproduce, as early as the 1980s and early 1990s [17]. The predictions of various selection system models, essentially based on the absence of radiographic lesions in breeding stock, forecast a significant reduction in the prevalence of lesions [34, 35]. However, various studbooks, faced with the complexity of the aetiopathogenesis of OCD, its dynamic aspect and the disappointing results of this selection, have reviewed their selection criteria and certain minor lesions are now accepted in male breeding stock [17]. However, to our knowledge, there is no longitudinal monitoring of the prevalence of OCD lesions in a population of warmblood horses.

The aim of this study was to evaluate changes in the prevalence of radiographic OCD lesions in a population of young sport horses in a given region over a 19-year period, taking into account the impact of breeding conditions (feeding and exercise) and sepsis on the development of lesions.

## Materials and methods

### Population

The study presented here is part of a large programme aimed at detecting developmental orthopaedic pathologies in horses born in Wallonia (Belgium). Several studies presenting the results of other aspects of the pathology have already been published [9, 18, 36–38]. The individuals were presented on the basis of a call for applications communicated by the studbooks as well as reminders of the existence of this programme via social media, website and studbook announcements. This programme was aimed at sport horse breeders in Wallonia, without restrictions on the size of the farm and its amateur or professional status, and consisted in carrying out radiographic examinations on foals from birth to 36 months. A document describing the procedure and objectives of the programme was given to each animal owner and to the treating veterinarian for approval before the individuals were examined. These examinations were conducted at the Centre européen du Cheval de Mont-le-Soie or at the breeder’s farm. All foals from the same breeding farm were systematically examined, within the limits of their cooperation.

All individuals included in this specific study had to be registered in a warmblood studbook: Anglo European Stud-book (AES), Belgian Warmblood Paard (BWP), Koninklijk Warmbloed Paard Nederland (KWPN), Hanoverian, SBS, Stud-book du Cheval de Selle Luxembourgeois (SCSL), Selle-Français (SF) and Zangersheide (Z). In addition, each individual had to be born in Wallonia, and the owner and veterinarian agreement document had to be completed.

### Radiographic examinations

All individuals with at least one radiographic examination carried out between the ages of 12 and 36 months were included.

A standardised radiographic protocol consisting of the following 12 views was used: latero-medial of the 4 fetlocks, latero-medial and plantarolateral-dorsomedial oblique of the hocks and latero-medial (or slightly caudolateral-craniomedial oblique) of the stifles.

If deemed by the operator, appropriate additional views, as described by Butler & al., were taken to confirm the presence or absence of lesions suspected during the standard radiographic protocol [16].To optimise radiographic examinations, the animals were sedated with Romifidine Hydrochloride (0.04mg/kg IV) or Detomidine Hydrochloride (0.01mg/kg) in combination with Butorphanol (0.02mg/kg IV).

X-ray images were generated using a Gierth RHF 200 ML portable device.

From 2006 to 2013, fluorescent screen cassettes were developed using a Vetray CR 2430 scanner and digital radiographs were examined using Vetray Vision version 4 image processing software (VetRay GmbH, Pfaffenhofen, Germany). From 2014 to 2020, the radiographs were developed using an Examion CR Vita 45 scanner and examined in digital format using Vita CR System Software V.3.2 (Carestream Health Rochester, NY, USA).

From 2021 to 2024, the radiographs were taken with the FUJIFILM Console Advance Software and sent to the VSOL programme for storage and reading.

### Radiographic interpretation

A veterinarian experienced in reading radiographs (RVC) carefully examined all the radiographs. All forms of OCD described in the literature were analysed. An OCD lesion was considered to be present when a fragment was observed near a joint margin. In the case of an oval area showing a delimited loss of density within the bone, the lesion was described as cystic. Variations in the appearance of the distal end of the medial trochlear ridge of the talus were considered anatomical variations and not lesions [39].

### Breeding conditions and anamnesis evaluation

For each individual x-rayed as part of this programme, a standardised questionnaire was distributed (S1 Appendix).

In the questionnaire, for each month since birth, the owner had to select one single type of housing condition from a table: permanent stabling alone (0), stabling alone with occasional outings (1), stabling alone with daily outings (2), stabling in groups with permanent access to the outdoors/permanent grassland (3) or closed stabling in group (4). With regard to feeding, given that the farmers were unable to give precise quantities of concentrate distributed, only a qualitative analysis, in four categories, was carried out: no concentrate (0), distribution of industrial concentrate (1), distribution of a mixture of home-made cereals (2), and distribution of a mixture of industrial concentrate coupled with a mixture of home-made cereals (3).

In addition, for each individual, an anamnesis was made from the breeder and from the questionnaire on the foal’s history (accidents, infections) since birth. In the questionnaire, the owner was asked to report the absence (0) or presence (1) of a septic phenomenon in the individual and, if present, to give a detailed description of the process and treatment (S1 Appendix).

### Statistical analysis

Palmar/plantar osteochondral fragments of the first phalanx (POFs) and subchondral cysts were not included in the prevalence calculations for the entire study population. In fact, it appears from the literature that POFs are more likely to be fragments of avulsion within ligament insertions, rather than resulting from a process of ischemic chondronecrosis [31, 40, 41]. As for cysts, a proportion of these are also more closely related to a traumatic process [15, 16].

With regard to breeding conditions, considering that breeding practices in Wallonia include a permanent outdoor lifestyle with grass for the vast majority of foals in the summer months, only the housing conditions and feeding practices during winter (at least 2 months) were evaluated for the statistical analysis. Each individual was, therefore, assigned to one of the 5 housing condition classes and 4 feeding categories described above.

In order to assess the impact of a septic phenomenon on the prevalence of OCD, individuals were classified in a "control" group when the owner did not indicate any infectious phenomenon in the individual (0) and in a "sepsis" group when a manifestation of infection was described (1). The prevalence of lesions was assessed in two ways: first by excluding the presence of POFs and then by including them in order to match previously published data [25].

The presence of a difference in the prevalence of OCD as a function of the X-ray device used, the housing conditions, the feeding practices, the studbook and the year of birth of the individual and its sires was evaluated by calculation of the Chi^2^ with a p-value< 0.05 using Microsoft Excel software and XLSTAT (Assinsoft, Paris, France). Given the low number of breeding individuals born in certain years, they were grouped together by groups born in 5 consecutive years.

Logistic regression using R-4.3.3 for Windows was performed to assess a correlation between the presence of OCD in the individuals and their year of birth, adjusted to the individuals’ housing and feeding conditions. Given the interaction between these two variables, for the logistic regression, they were combined to create a variable integrating both the type of diet (0, 1, 2, 3 or unknown) and the accommodation of the individual (0, 1, 2, 3, 4 or unknown).

Given the previous publications on the effects of diet and accommodation on the presence of OCD, an in-depth analysis of these factors was not carried out as part of this study [18, 36].

## Results

A group of 1099 individuals born between 2004 and 2022 was studied. These included 525 females and 574 males. The distribution within the studbooks was as follows: 669 SBS, 105 BWP, 104 Zangersheide, 96 SF, 63 Hanoverian, 40 AES, 20 SCSL and 2 KWPN.

**Fig 1:**
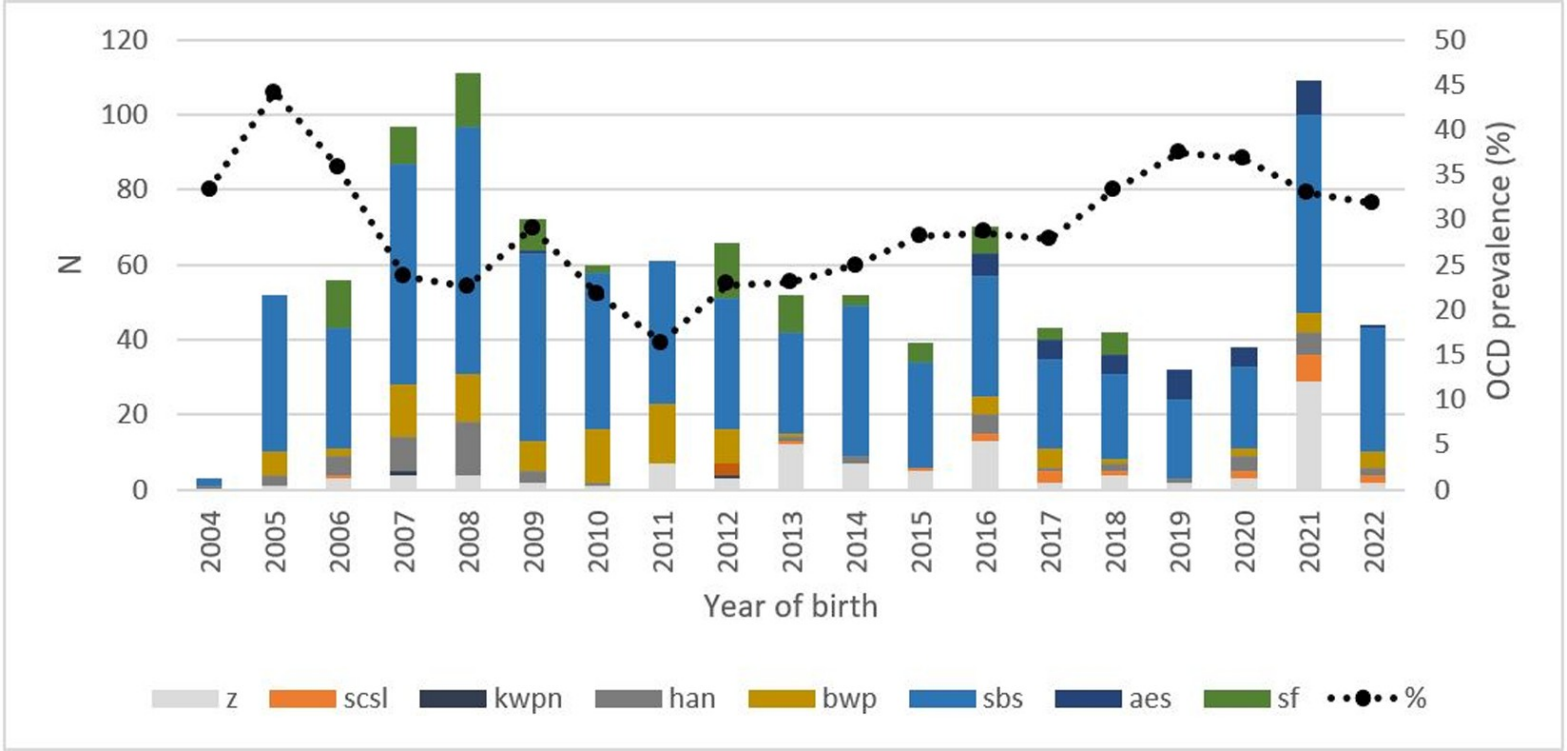
Evolution of the number of individuals radiographed by studbook and of the prevalence of OCD lesions in Walloon sport horses born from 2004 to 2022. N = Number of individuals radiographed by year and studbook; Z = Zangersheide; SCSL = Studbook du Cheval de Selle Luxembourgeois; KWPN = Koninklijk Warmbloed Paard Nederland; Han = Hanoverian; BWP = Belgian Warmblood Paard; SBS = Stud-book du Cheval de Sport Belge; AES = Anglo European Stud-book; SF = Selle-Français.

Upon radiographic examination, carried out from November 2006 to March 2024, the mean age was 644 days (SD: 105.3 days; max: 1055 days, min. 365 days). A group of 309 individuals (28.1%) had at least one OCD lesion. The number of individuals radiographed and the percentage of horses with OCD per year are expressed in Fig 1.

**Fig 2:**
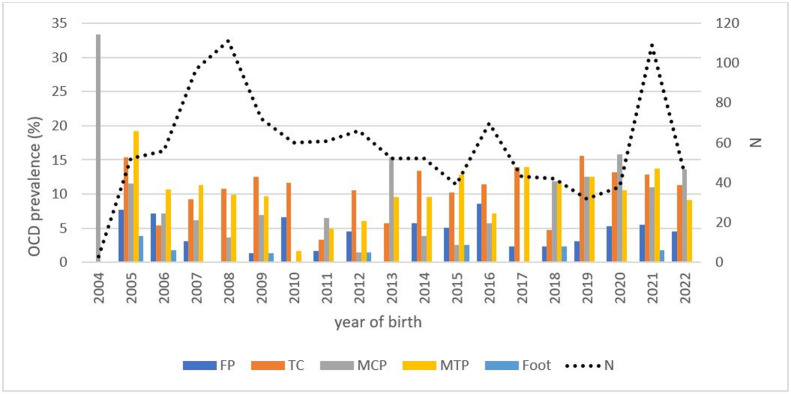
OCD prevalence by articular type and number of Warmblood horses born in Wallonia between 2004 and 2022. FP = Femoropatellar; TC = Tarsocrural; MCP = Metacarpophalangeal; MTP = Metatarsophalangeal; N = number of horses radiographied/year. Individuals in the BWP studbook had significantly fewer lesions (18/105) than those in the Zangersheide studbook (41/104) (p < 0.05). No other significant difference could be demonstrated based on studbooks.

With regard to the distribution of lesions within the various joints (Fig 2), 9 individuals (0.8%) had fragments of the extensor process of the third phalanx in the forefeet (8 unilateral, 1 bilateral,), 79 (7.2%) had OCD of the metacarpophalangeal joints (70 unilateral, 9 bilateral). The distribution within this joint was as follows: 46 Dorso-proximal fragments of the first phalanx and 42 fragments of the third metacarpal bone sagittal crest. In addition, 12 POFs were observed (all unilateral) and were not included in the OCD prevalence.

Concerning the metatarsophalangeal joints, 111 individuals (10.1%) had OCD (91 unilateral, 20 bilateral). There were 48 Dorso-proximal fragments of the first phalanx and 84 sagittal crest fragments. In addition, 63 individuals had POFs (56 unilateral, 7 bilateral).

In the hock, 116 individuals (10.6%) had at least one osteochondral fragment (84 unilateral, 32 bilateral). Of these, 107 were from the intermediate ridge of the tibial cochlea, 32 from the lateral trochlear ridge of the talus, and 10 from the medial trochlear ridge of the talus.

A group of 44 individuals (4%) had at least one femoropatellar OCD lesion, 12 of which were bilateral lesions of the lateral trochlear ridge of the femur, and the rest (32) were unilateral.

**Fig 3:**
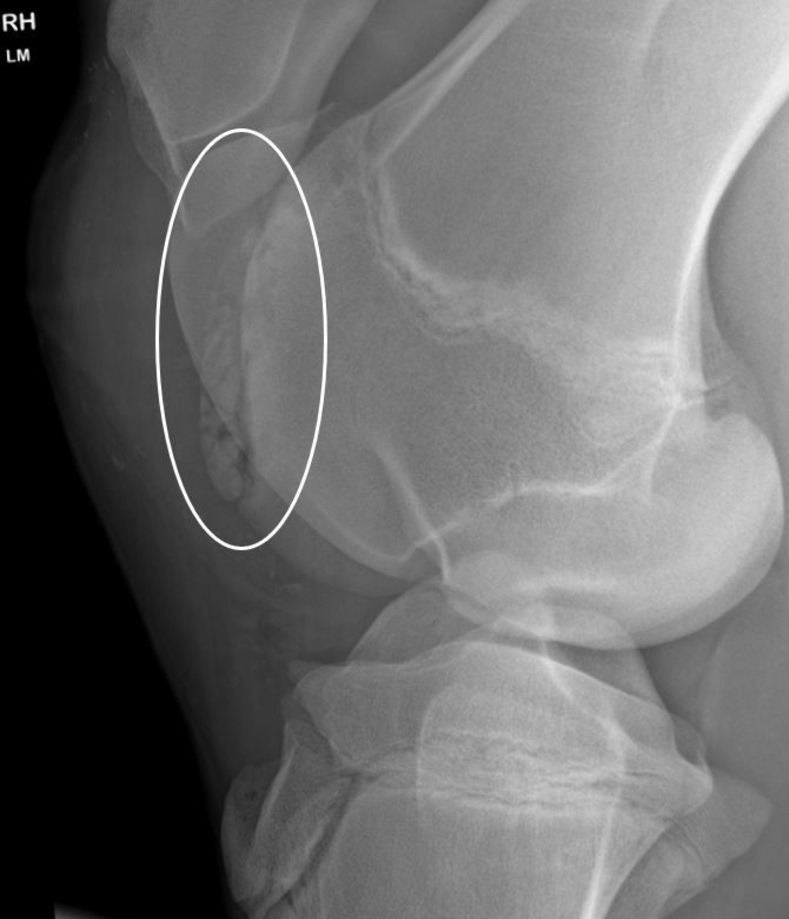
Latero-medial radiography of the right stifle of a horse which suffered from omphalophlebitis in the neonate period and was treated in the field. There is an extensive osteochondrosis dissecans lesion of the lateral trochlear ridge (white circle) associated with severe distension of the femoropatellar joint.

In the questionnaires of 385 individuals, the owners described whether or not sepsis was present. Among these, a septic process was suspected or had been observed in 21 (5.5%) individuals, while in 364 (94.5%) individuals no such phenomenon was described (S1 Table). The prevalence of OCD lesions in the latter group was 28% (103/364), whereas 14 individuals (67%) in the "sepsis" group were affected, which was significantly higher (p = 0.0007) (Fig 3). In addition, 4 individuals with sepsis had POFs. When this type of fragment was included in the prevalence of OCD, the prevalence of osteochondral fragments was 17/21 (81%) in the sepsis group and 118/364 (32.4%) in the control group.

The sires of the individuals included in this study were born between 1977 and 2018, and the mothers between 1982 and 2018. When the individuals were born, the average age of their fathers was 11.5 years and 11 years for their mothers. There was no significant correlation between the date of birth of the breeding individuals (grouped into groups born in 5 consecutive years) and the prevalence of OCD in the individuals radiographed (sires: p = 0.102; mares: p = 0.63).

With regard to the radiography system used, 546 (147 OCD), 338 (90 OCD) and 215 (72 OCD) individuals were radiographed with the Vetray CR 2430, Examion CR Vita 45 and FUJIFILM Console Advance Software radiography systems respectively. The prevalence of OCD lesions observed was not significantly different according to the device used (p = 0.148).

Information concerning housing and feeding practices was available for 755 and 752 individuals respectively. Housing and feeding practices were significantly (p<0.05) correlated with the presence of OCD.

Finally, no significant correlation was observed in the univariate analysis between individuals’ year of birth and the prevalence of OCD lesions (p = 0.181). In the logistic regression, the year of birth of the individuals was not significantly correlated with the presence of OCD (p = 0.259).

## Discussion

The results of this study do not demonstrate a reduction in the prevalence of osteochondrosis dissecans in the population of Belgian horses born between 2004 and 2022 and radiographed as part of this programme. Indeed, the total prevalence of individuals with OCD was 28.1%, which is in line with previous studies, and the trend was upwards, with 26.9% of individuals affected between 2006 and 2013, 26.6% between 2014 and 2020, and 33% between 2021 and 2024 (all three periods correspond to changes in the X-ray systems used) [6, 42].

Given that the heritability (h^2^) of osteochondrosis lesions has frequently been estimated to be greater than 0.25, OCD is assumed to be sensitive to selection programmes based on the absence of radiographic lesions in breeding stock [17, 30, 43]. Since the 80s and 90s, the femoropatellar and tarsocrural joints have been X-rayed as part of stallion expertise of several studbooks, and males showing OCD have been systematically excluded from breeding programmes. In fact, studies shaped the expected results based on different strong selection methods leading to a significant decrease in the prevalence of lesions [34, 35]. In this study, although all individuals were registered in studbooks for which only males without lesions typical of the osteochondrosis process were allowed to breed, the results did not demonstrate a significant reduction in the prevalence of lesions in this population. This is also consistent with the observations made by the KWPN studbook after years of selecting stallion candidates free of lesions, observations that led to an adaptation of their selection criteria against OCD. In recent years, males with minor lesions have been able to be included in KWPN breeding programmes. Breeding values have been calculated and, if the progeny of these stallions have a high rate of OCD, they can be eliminated as breeding stock [17].

Several theories may explain these results. Firstly, it is possible that the selection criteria were not strong enough to obtain a significant reduction in the prevalence of OCD over one or two generations. In fact, given that the average age of the sires was 11.5 years, this study corresponds to one or two generations of selection of males without OCD lesions. The term ’without OCD’ should be taken with caution, as with some lesions, osteochondral fragments may have been removed prior to stallion selection and only the joints most frequently affected were examined. Furthermore, mares with OCD are not excluded from selection programmes. Secondly, among the aetiological factors of osteochondrosis, it seems that a proportion of the fragments have only a limited hereditary origin, and biomechanical and dietary factors are thought to be involved in their formation [20, 30, 44, 45]. Consequently, the inclusion of these fragments in prevalence calculations would limit the observed effect of selection.

Even within fragments with a proven hereditary origin, their dynamics are sensitive to breeding conditions [18, 19, 23, 36, 45, 46]. The results presented here confirm that the feeding and housing practices of individuals influence the presence of OCD. Consequently, in order to reduce the prevalence of lesions, it seems necessary to make greater efforts to raise farmers’ awareness of good breeding practices in order to reduce its appearance and promote the regression of lesions. These breeding practices particularly concern housing conditions, by favouring moderate and regular exercise for individuals throughout their growth, and feeding, by limiting cereal intake while maintaining an adequate mineral supply [18, 19, 21, 24, 47].

In addition, given that the expression of a number of genes is altered in individuals with OCD and that studies on DNA strand sequence modifications yield heterogeneous results, studies investigating the interactions between genetics and the environment should be considered [43, 48, 49]. Among the mechanisms involved, epigenetic modifications-referring to heritable changes in the expression of genes without alteration of the DNA sequence itself-, involved notably in the process of enchondral ossification, should be considered in the future [50]. These epigenetic mechanisms include alterations in microRNAs, histone and DNA methylation processes and metabolic pathways in response to environmental stimuli, which have been shown to play a potential role in the development of various pathologies, including osteochondrosis dissecans [50–52].

This study also demonstrates that the presence of sepsis during the foal and yearling periods is a significant factor in the development of OCD and POFs. It is the first study with a control group to report this. Previously, the team of Hendrickson & colleagues had demonstrated a 67.9% prevalence of osteochondral fragments in fetlocks and hocks in a cohort of 28 standardbred foals hospitalised and surviving an infection before the age of 6 months [25]. Wormstrand et al subsequently demonstrated the presence of bacteria in the cartilage canals of the growth cartilage and the physis as well as neutrophils in 7 foals euthanised for septic arthritis/osteomyelitis [26]. This study confirms the hypothesis that the presence of infection in foals and yearlings, a period during which the dynamics of osteochondrosis lesions are active, is a significant risk factor [38, 41, 53–55]. It also supports the idea that infections that have not led to hospitalisation are likely to favour the appearance of osteochondral fragments, notably at femoropatellar level, which had not previously been observe. Furthermore, individuals in the ’sepsis’ group were particularly prone to developing POFs, with 4/21 (19%) affected individuals compared with 23/364 (6.3%) in the ’control’ group. This suggests that an infectious origin could also be at the origin of these fragments, generally considered to be traumatic [40, 41, 56]. Individuals which suffered from sepsis during the growing period and OCD should therefore not necessarily be excluded from breeding given the lack of diagnostic tools to differentiate between lesions of septic cause and those inherent in the presence of genes predisposing to OCD.

### Limitations of the study

This study has a number of limitations. Firstly, the diagnostic method used and the interpretation of the radiographs. Although, in this study, the bias linked to inter-observer sensitivity in the detection of radiographic lesions was ruled out by the fact that all examinations were read by a single operator, a bias linked to the imaging system used and the operator who performed the examination could not be ruled out [57–59]. Although no significant correlation between the prevalence of OCD and the radiography system used was detected (p = 0.148), the prevalence of OCD lesions was 26.9%, 26.6% and 33% with two CR (Computed Radiography) and one DR (Direct Radiography) systems respectively. Developments in technology have not only improved the quality of each image, but have also made it easier to take X-rays. It has been shown that increasing the number of radiographic views increases the sensitivity of OCD lesion detection [15, 57, 58]. Thus, the difference observed in the prevalence of lesions between the CR and DR systems may have more to do with the system used than with a *per se* change in the prevalence of lesions.

Furthermore, the prevalence of OCD observed in this study certainly underestimates the true prevalence in the population studied. Firstly, the standardised radiographic protocol of this study only includes certain types of joints, whereas osteochondrosis lesions may be present in virtually all diarthrodial joints [17, 24, 60–62]. Secondly, radiography offers limited sensitivity in the detection of OCD lesions, which also depends on the operator and their experience [57–59]. Finally, certain lesions have not been voluntarily included in the prevalence calculations due to their uncertain aetiology, which is notably the case for cystic lesions of the first phalanx, POFs and osteochondral fragments of the proximal sesamoid bones [15, 16, 40, 44].

Sampling bias cannot be ruled out. Individuals were recruited on a voluntary basis and all individuals from the same farm were systematically radiographed. However, farms with a high prevalence of developmental orthopaedic pathologies may have been unintentionally selected. In order to eliminate any sampling bias, the selection of individuals should be randomised.

With regard to the correlation between OCD and sepsis, given that the diagnosis of infection was made on the basis of an anamnesis from the breeder and a questionnaire, some individuals were included in the "sepsis" group when an infectious phenomenon was only suspected. In order to carry out a precise study of the impact of sepsis on the prevalence of OCD, it would be necessary to precisely target the type of infectious process and implement a diagnostic tool to confirm it. Furthermore, a possible effect of the treatment administered on the development of OCD could not be assessed in this group and further studies are required to evaluate this parameter.

In conclusion, this study shows a limited change in the prevalence of OCD lesions in the population of Warmblood horses born in Wallonia between 2004 and 2022. In order to reduce the prevalence of this pathology, a change in breeding practices should be associated with the selection of lesion-free breeding individuals, while not necessarily excluding those that have suffered from bacterial infection during the growing period and have OCD lesions.

## Supporting information

S1 Appendix Document for collecting anamnesis and breeding conditions of individuals. (PDF)

S1 Table Description of the septic process and associated treatment of the 21 individuals for whom an infectious phenomenon was reported. SR: Sagittal Ridge; LTRT: Lateral trochlear ridge of the talus; DPP1: Dorso-Proximal of the first phalanx; DIRT:Distal Intermediate Ridge of the tibia; POF: Palmar/plantar Osteochondral Fragment.
(ODS)

## Abbreviations

OCD: Osteochondrosis dissecans
POF: Palmar/plantar osteochondral fragment
