# Supplementary material for: Nineteen years of radiographic screening: Impact of sepsis and evolution of osteochondrosis dissecans prevalence in Walloon sport horses born between 2004 and 2022
Source: PLoS One. 2024 Sep 10;19(9):e0308304. doi: 10.1371/journal.pone.0308304 (PMC11386463; doi:10.1371/journal.pone.0308304)
Supplement: S1 Appendix — (PDF) [file pone.0308304.s001.pdf]

# Questionnaire épidémiologique

*Etude sur l'ostéochondrose dissécante et autres pathologies orthopédiques du développement chez les chevaux de sport.*

**Merci pour le temps que vous voudrez bien consacrer à remplir ce formulaire de manière la plus complète possible.**
**Les données récoltées resteront confidentielles et les résultats publiés de cette étude ne seront d'aucune manière associés à votre nom.**

---

## 1. Informations sur PROPRIETAIRE :

| | |
|---|---|
| **Nom et prénom** | |
| **Adresse** | |
| **Code postal** | |
| **Localité** | |
| **Numéro de tél. / GSM** | |
| **Fax** | |
| **E-mail** | |
| **Vétérinaire traitant** | |
| **Marechal** | |

Formulaire à renvoyer à : **Rousset Alycia**
**Centre Européen du Cheval**
**Mont-le-Soie, 1**
**6690 Vielsalm**
**Tél. : +3280216556**
**Mail : alycia.rousset@montlesoie.be**

## 2. Informations sur le POULAIN

| | |
|---|---|
| **Nom** | |
| **Stud-book et numéro** | |
| **Sexe** | |
| **Date de naissance** | |
| **Père** | |
| **Mère** | |
| **Père de mère** | |
| **N° de puce** | |
| **Destination** | Exclu - Maintenu dans la chaîne alimentaire |

- Votre poulain a-t-il déjà eu des problèmes locomoteurs (gonflement, gêne, accident, blessure, ...) diagnostiqués par *vous* ou *par un vétérinaire* (à quel âge, de quel type,...) ? **oui / non**
  ..........................................................................................................................
  ..........................................................................................................................

- Avez-vous déjà fait des radios sur le poulain ? **oui / non**
  Pourquoi ?
  ..........................................................................................................................
  ..........................................................................................................................

- A-t-il eu des problèmes d'autre nature ? **oui / non**...........................................................
  .....................................................................................................................................
  ...............................................................................................................

- A-t-il déjà reçu des traitements médicamenteux (pour quels motifs, à quel âge, de quelle nature, à quelle dose, durant combien de temps,...) ? **oui / non**
  .....................................................................................................................................
  ...............................................................................................................

- Reçoit-il, ou a-t-il reçu des promoteurs de croissance ? **oui / non**
  Si oui, de quel type, de quelle manière ?...........................................................................
  ..........................................................................................................................

- A quelle date votre cheval a-t-il été sevré ? Par quelle méthode (abrupt, progressif, autre décrivez) ?
  .........................................................................................................................

- Quels sont vos critères de sevrage :

  âge du poulain – saison – météo – embonpoint – gestion par lots – santé de la mère....

- Comment estimez-vous l'embonpoint de votre cheval :

  trop maigre – maigre – normal(-) – normal(+) – gras – trop gras

- A quel âge votre cheval a-t-il été paré pour la première fois ? ...........................................
  A quelle fréquence ensuite est-il paré ? ..............................................................................

| **POULAIN** : **Alimentation** | **Janv** | **Fév** | **Mars** | **Avril** | **Mai** | **Juin** | **Juill** | **Aout** | **Sept** | **Oct** | **Nov** | **Déc** |
|---|---|---|---|---|---|---|---|---|---|---|---|---|
| Uniquement lait maternel | | | | | | | | | | | | |
| | | | | | | | | | | | | |
| Partage la ration de sa mère | | | | | | | | | | | | |
| | | | | | | | | | | | | |
| Herbe | | | | | | | | | | | | |
| | | | | | | | | | | | | |
| Mélange tout fait :<br>Marque<br>Quantité (préciser en litre ou en kilo) | | | | | | | | | | | | |
| | | | | | | | | | | | | |
| Mélange fait par vous :<br>Sorte<br>Quantité (préciser en litre ou en kilo) | | | | | | | | | | | | |
| | | | | | | | | | | | | |
| Compléments aliment :<br>Marque<br>Fréquence | | | | | | | | | | | | |
| Pierre à lécher : marque ou type | | | | | | | | | | | | |
| Foin / Quantité :<br>Qualité : B – TB | | | | | | | | | | | | |
| Préfané / Quantité | | | | | | | | | | | | |

**REMARQUES** :

| **b) Management :** | **Janv** | **Fév** | **Mars** | **Avril** | **Mai** | **Juin** | **Juill** | **Aout** | **Sept** | **Oct** | **Nov** | **Déc** |
|---|---|---|---|---|---|---|---|---|---|---|---|---|
| Au boxe en permanence<br>Taille du boxe | | | | | | | | | | | | |
| | | | | | | | | | | | | |
| Au boxe et lâché en piste occasionnellement<br>Taille du boxe | | | | | | | | | | | | |
| | | | | | | | | | | | | |
| Au boxe tout le temps et lâché en piste quotidiennement ou presque<br>Taille du boxe | | | | | | | | | | | | |
| | | | | | | | | | | | | |
| Principalement au boxe, occasionnellement en prairie<br>Taille du boxe | | | | | | | | | | | | |
| | | | | | | | | | | | | |
| En prairie la journée, au boxe la nuit<br>Taille du boxe | | | | | | | | | | | | |
| | | | | | | | | | | | | |
| En prairie en permanence | | | | | | | | | | | | |
| | | | | | | | | | | | | |
| Seul (ou avec sa mère) | | | | | | | | | | | | |
| | | | | | | | | | | | | |
| Avec d’autres poulains - chevaux | | | | | | | | | | | | |
| | | | | | | | | | | | | |
| Quelle est son degré d’activité :<br>paisible – actif – excité | | | | | | | | | | | | |
| | | | | | | | | | | | | |

Si au boxe : paille - copeaux - Autres (préciser) :

**REMARQUES** :

# 3. Informations sur la MERE

## A. Infos générales:

- S'agit-il d'une mère porteuse ? **oui / non** (Si oui, le questionnaire porte sur la mère-porteuse)
- Age de la mère à la naissance de ce poulain : ...............................................................
- La mère biologique , a-t-elle un statut OCD positif ? **oui / non**
- Nombre de gestations de la mère (y compris ce poulain-ci) : .......................................
- Etait-elle suitée au moment de la saillie ? **oui / non**
- Si oui, à quel stade de la gestation le poulain précédent a-t-il été sevré ? .......................
- Par quelle méthode le poulain précédent a-t-il été sevré (abrupt, progressif, autre décrivez) ? …………………………………………………………………………………………………………… ……………………………………………
- A-t-elle été re-saillie pendant la lactation de ce poulain-ci ? **oui / non**
- Si oui, à quel âge du poulain a-t-elle été inséminée avec succès ?.................................
- Insémination en frais ou congelé ? (biffer la mention inutile)
- Pour quelle(s) raison(s) la jument a-t-elle été mise à la reproduction ?
  - ☐ Elevage
  - ☐ Accident (nature de l'accident)........................................................................
  - ☐ Bref arrêt dans une carrière de travail :
    - Durée de l'arrêt : ........................................................................................
    - A quel mois de gestation le travail de la jument a-t-il été arrêté avant le poulinage ?.............................................................................................

## B. Antécédents :

- La mère a-t-elle déjà eu des problèmes locomoteurs (gonflement, gêne, accident, blessure, ...) diagnostiqués par vous ou *par un vétérinaire* (à quel âge, de quel type,...) ? .................................................................................................................... .................................................................................................................... ....................................................................................................................
- A-t-elle eu des problèmes d'autre nature ? .................................................................................................................... ....................................................................................................................
- La mère a-t-elle déjà fait l'objet de radiographies ou d'autres examens (raisons et diagnostic) ? .......................................................................................................... . .................................................................................................................. ....................................................................................................................
- Les parents directs de votre cheval (frères-sœurs) ont-ils déjà eu des problèmes locomoteurs (de quels types, à quel âge, ...) ? .................................................................................................................... .................................................................................................................... ....................................................................................................................
- Les parents ont-ils été présentés à une visite d'achat ou à une expertise vétérinaire (à quel âge, résultats) ? ..........................................................................................

## **Mère :** **de la saillie au sevrage**

| **Alimentation** | **Janv** | **Fév** | **Mars** | **Avril** | **Mai** | **Juin** | **Juill** | **Aout** | **Sept** | **Oct** | **Nov** | **Déc** |
|---|---|---|---|---|---|---|---|---|---|---|---|---|
| Herbe | | | | | | | | | | | | |
| | | | | | | | | | | | | |
| Mélange tout fait :<br>Marque<br>Quantité (préciser en litre ou en kilo) | | | | | | | | | | | | |
| | | | | | | | | | | | | |
| Mélange fait par vous :<br>Sorte<br>Quantité (préciser en litre ou en kilo) | | | | | | | | | | | | |
| | | | | | | | | | | | | |
| Compléments aliment :<br>Marque<br>Fréquence | | | | | | | | | | | | |
| Pierre à lécher : marque ou type | | | | | | | | | | | | |
| Foin / Quantité :<br>Qualité : B – TB | | | | | | | | | | | | |
| Préfané / Quantité | | | | | | | | | | | | |

**REMARQUES** :

## **Mère :** de la saillie au sevrage

| **b) Management :** | **Janv** | **Fév** | **Mars** | **Avril** | **Mai** | **Juin** | **Juill** | **Aout** | **Sept** | **Oct** | **Nov** | **Déc** |
|---|---|---|---|---|---|---|---|---|---|---|---|---|
| Au boxe en permanence<br>Taille du boxe | | | | | | | | | | | | |
| | | | | | | | | | | | | |
| Au boxe et lâché en piste occasionnellement<br>Taille du boxe | | | | | | | | | | | | |
| | | | | | | | | | | | | |
| Au boxe tout le temps et lâché en piste quotidiennement ou presque<br>Taille du boxe | | | | | | | | | | | | |
| | | | | | | | | | | | | |
| Principalement au boxe, occasionnellement en prairie<br>Taille du boxe | | | | | | | | | | | | |
| | | | | | | | | | | | | |
| En prairie la journée, au boxe la nuit<br>Taille du boxe | | | | | | | | | | | | |
| | | | | | | | | | | | | |
| En prairie en permanence | | | | | | | | | | | | |
| | | | | | | | | | | | | |

**REMARQUES** :

## 3. Gestion des PATURES

- Enrichissez-vous vos prairies ? **oui / non**

  Quel type d'engrais ? ……………………………………………………………….........................

  A quelle dose,  fréquence / période ? ......................................................................

  ..............................................................................................................................

- Traitez-vous vos prairies (insecticides, chaux, cyanamide, autre… ) ?

  Quel type de produits ? ………………………………………………………….........................

  ……………………………………………………………………………………….........................

  A quelle dose,  fréquence / période ? ......................................................................

  ……………………………………………………………………………………….........................

- Fanez-vous du foin sur vos prairies avant de pâturer les poulains ? **oui / non**

  A quelle saison ? …………………………………………………………............................

- Ebousez-vous vos prairies ? **oui / non**        Fréquence : ..........................................

- La pâture, et/ou le paddock, est-elle :

  - ☐ Plate
  - ☐ Vallonnée
  - ☐ Abrupte

  - ☐ Bien herbagée
  - ☐ Plutôt rase ou en terre
  - ☐ Boueuse
  - ☐ Caillouteuse

- Nombre de chevaux à l'Ha :

- Comment qualifieriez-vous la qualité de l'herbe ?

  …………………………………………………………………………….....................

- Comment qualifieriez-vous le sol de vos prairies (schisteux, argileux, sablonneux, ...)

  ………………………………………………………………………….......................

- Avez-vous déjà fait une analyse du sol de vos pâtures ? **oui / non**

  Résultats :

## 4. Remarques éventuelles.
